# Deiodinase Knockdown during Early Zebrafish Development Affects Growth, Development, Energy Metabolism, Motility and Phototransduction

**DOI:** 10.1371/journal.pone.0123285

**Published:** 2015-04-09

**Authors:** Enise Bagci, Marjolein Heijlen, Lucia Vergauwen, An Hagenaars, Anne M. Houbrechts, Camila V. Esguerra, Ronny Blust, Veerle M. Darras, Dries Knapen

**Affiliations:** 1 Systemic Physiological and Ecotoxicological Research (SPHERE), Department of Biology, University of Antwerp, B-2020 Antwerpen, Belgium; 2 Zebrafishlab, Veterinary Physiology and Biochemistry, Department of Veterinary Sciences, University of Antwerp, B-2160 Wilrijk, Belgium; 3 Laboratory of Comparative Endocrinology, Animal Physiology and Neurobiology Section, Department of Biology, KU Leuven, B-3000 Leuven, Belgium; 4 Laboratory for Molecular Biodiscovery, Department of Pharmaceutical and Pharmacological Sciences, KU Leuven, B-3000 Leuven, Belgium; University Claude Bernard Lyon 1, FRANCE

## Abstract

Thyroid hormone (TH) balance is essential for vertebrate development. Deiodinase type 1 (D1) and type 2 (D2) increase and deiodinase type 3 (D3) decreases local intracellular levels of T_3_, the most important active TH. The role of deiodinase-mediated TH effects in early vertebrate development is only partially understood. Therefore, we investigated the role of deiodinases during early development of zebrafish until 96 hours post fertilization at the level of the transcriptome (microarray), biochemistry, morphology and physiology using morpholino (MO) knockdown. Knockdown of D1+D2 (D1D2MO) and knockdown of D3 (D3MO) both resulted in transcriptional regulation of energy metabolism and (muscle) development in abdomen and tail, together with reduced growth, impaired swim bladder inflation, reduced protein content and reduced motility. The reduced growth and impaired swim bladder inflation in D1D2MO could be due to lower levels of T_3_ which is known to drive growth and development. The pronounced upregulation of a large number of transcripts coding for key proteins in ATP-producing pathways in D1D2MO could reflect a compensatory response to a decreased metabolic rate, also typically linked to hypothyroidism. Compared to D1D2MO, the effects were more pronounced or more frequent in D3MO, in which hyperthyroidism is expected. More specifically, increased heart rate, delayed hatching and increased carbohydrate content were observed only in D3MO. An increase of the metabolic rate, a decrease of the metabolic efficiency and a stimulation of gluconeogenesis using amino acids as substrates may have been involved in the observed reduced protein content, growth and motility in D3MO larvae. Furthermore, expression of transcripts involved in purine metabolism coupled to vision was decreased in both knockdown conditions, suggesting that both may impair vision. This study provides new insights, not only into the role of deiodinases, but also into the importance of a correct TH balance during vertebrate embryonic development.

## Introduction

Thyroid hormones (THs) play an important role in a wide range of biological processes in vertebrates including growth, development, reproduction, cardiac function, thermoregulation, response to injury, tissue repair and homeostasis [1–4]. To date, three deiodinases (D) have been characterized that locally activate or inactivate THs and are therefore important mediators of TH action. Both D1 and D2 are able to convert the pro-hormone thyroxine or T_4_ into the receptor-active hormone 3,5,3’-triiodothyronine or T_3_, whereas D3 catalyzes the conversion of T_4_ to rT_3_ (nuclear receptor-inactive form) and T_3_ to 3,3’-T_2_, which is biologically inactive [5–7]. In our previous work, as well as in many other studies, it has been reported that deiodinases play an essential role in vertebrate development by regulating local TH levels during crucial stages of embryonic development [8–12].

In recent years, the zebrafish (*Danio rerio*) embryo has become a valuable model of vertebrate development with many practical advantages, making it an excellent model for studying the role of deiodinases in vertebrate embryonic development. We and others recently showed that zebrafish embryos express two isoforms of D3, encoded by *dio3a* and *dio3b*, and that the contribution of *dio3b* to embryonic D3 activity is predominant [10,13]. In this study, zebrafish embryos were microinjected into the yolk with morpholinos (MOs) directed against D1,D2 and D3 (*dio3b*) or with standard control MO to produce D1D2MO (combination of deiodinase type 1 and type 2 morpholino-injected), D3MO (deiodinase type 3 morpholino-injected), and SCMO (standard control morpholino-injected) groups. We previously showed that knockdown of D1 alone seemed to have little effect on zebrafish early development. In contrast, D2 knockdown resulted in a developmental delay, and combined knockdown resulted in a more pronounced delay and additional morphological effects, showing that D1 may only be crucial in a state of hypothyroidism [14]. Therefore we opted to use a combined D1D2 knockdown to effectively block the complete thyroid hormone activating pathway. Additionally, our previous studies showed that both D2MO and D1D2MO phenotypes could be largely rescued by T_3_ supplementation [11,14] and that the phenotype of D3MO zebrafish embryos could be partially mimicked by T_3_ supplementation and rescued by injection of human D3 mRNA [10]. Although it was not possible to actually measure T_3_ and T_4_ levels in these studies, this strongly suggests that D1D2MO suffer from hypothyroidism and D3MO from hyperthyroidism. Deiodinase knockout mice were shown to balance T_3_ levels by regulating T_4_ and TSH levels [15]. Mouse embryos can benefit from maternal TH homeostasis during gestation *in utero*. However, in oviparous species, embryos fully rely on a fixed amount of maternal THs deposited in the yolk until activation of the embryonic thyroid; for zebrafish this occurs around 72 hpf (hours post fertilization) [16]. Already during early development, deiodinases are essential for the activation of maternally transferred TH. Therefore, this early developmental period is the focus of the current study, and the transcriptional responses are investigated at 72 hpf.

The mechanisms through which deiodinases regulate embryonic development are currently unclear. Full genome transcriptional profiling to elucidate underlying molecular events in deiodinase-deficient zebrafish embryos has never been applied before. Since deiodinases are known to have tissue-specific expression patterns [17], we microdissected early larvae (72 hpf) into head, abdomen and tail regions. We subsequently analyzed transcriptional regulation in D1D2MO and D3MO in these three body regions, using full genome microarrays. In order to relate transcriptional effects to phenotypic effects, we studied the phenotypes of D1D2MO and D3MO embryos and larvae at different developmental time points spanning from 24 hpf to 96 hpf.

For the first time, we have analyzed the transcriptional response of deiodinase knockdowns in specific embryonic regions. This study therefore provides a useful resource for future research aimed at understanding the role of deiodinases and THs in different body regions/tissues of early developmental life stages.

## Material and Methods

### 1. Zebrafish handling and embryo collection

Adult fish were kept and microinjections were carried out at the KU Leuven. Wild Indian Karyotype (WIK) zebrafish were maintained in 80 L community tanks, containing biologically filtered, medium-hard reconstituted freshwater (OECD ISO-6341-1982, 290 mg/L CaCl_2_.2H_2_O, 120 mg/L MgSO_4_.7H_2_O, 60 mg/L NaHCO_3_, 6 mg/L KCl) at 28 ± 1°C under a light/dark regime of 14/10 h. The breeding stock was fed twice a day, once with live brine shrimp larvae (*Artemia*, raised up from cysts which were purchased from Ocean Nutrition Europe) and once with formulated food (Dr. Bassleer Biofish food, Telgte, Germany). Embryos were produced by natural mating and spawning of 6- to 9-month-old fish, and were collected within 20 minutes post fertilization.

#### Ethics statement

This study was approved by the Institutional Ethical Committee of the KU Leuven (project number P102/2012) and experiments were executed in strict accordance with the European Council Directive (2010/63/EC). According to the EU Directive on the protection of animals used for scientific purposes (2010/63/EU) and the Commission Implementing Decision (2012/707/EU), fish are non-protected animals until the stage of free feeding; this limit was set at 120 hpf for zebrafish. The experiments in this study lasted only up to 96 hpf.

### 2. Microinjection of morpholinos: deiodinase knockdown

An antisense oligonucleotide MO knockdown approach was used to transiently block translation of T_3_ producing (D1+D2) or inactivating (D3) deiodinases. Sequences for SCMO, D1MO and D2MO were taken from Walpita et al. [14]. The sequence for D3MO (*dio3b*) was taken from Heijlen et al. [10]. All MOs were purchased from GeneTools (LLC, Philomath, OR). In total, three MO solutions, namely SCMO, D1D2MO (a combination of D1MO and D2MO) and D3MO, were prepared in RNAse- and DNase-free Gibco-water (Life Technologies, Gent). Based on preliminary experiments, the optimal concentration of the MOs was determined to be 0.4 mM (0.2+0.2 mM in the case of D1D2MO) and the injection volume 2 nl as described earlier in detail [10]. This volume was injected into the yolk of 2- to 4-cell stage embryos, resulting in a delivery of approximately 6.6 ng MO per embryo. The specificity of the used D1D2MO and D3MO was demonstrated previously using rescue experiments with mRNA injection and/or T_3_ supplementation [10,11,18]. MO solutions were microinjected using a Femtojet microinjector (Eppendorf, Belgium) and a M3301 micromanipulator (World Precision Instruments, UK). To control delivery efficiency of the MO, each MO solution was combined with phenol red dye (final percentage of 0.15%). After microinjection, embryos were raised at 28 ± 0.5°C in 0.3x Danieau’s solution (prepared from 30x stock solution: 1,74 M NaCl, 21 mM KCl, 12 mM MgSO_4_.7H_2_O, 18 mM Ca[NO_3_]_2_.4H_2_O and 150 mM HEPES, pH 7.6).

### 3. Microarray analysis

#### Microdissection and RNA isolation

For each condition, embryos were reared together in a Petri dish (PS, 90 x 16.2 mm, MLS NV, Belgium) with a maximum number of 40 embryos. Using a stereomicroscope (M4A, Wild Heerbruggbino) and a pair of #55 Dumont forceps (Fine Tools, Berlin), 72 hpf-old zebrafish larvae were manually dissected in ice-cold RNAse-free water to separate the head, abdomen and tail regions. The head region contained the brain, sacculi/otolith and jaw parts, the abdomen region contained the yolk, swim bladder, liver and the entire gastrointestinal tract, and the tail region consisted of the remainder reaching to the tail tip. Four biological replicates of each knockdown condition (SCMO, D1D2MO, D3MO) and each body region were collected. A total of 20 head, 40 abdomen and 40 tail fragments were pooled per replicate in an RNAse-free Eppendorf tube and immediately snap frozen on dry ice. Samples were kept at -80°C until processing for RNA extraction.

Total RNA was extracted from the tissue pools using Qiazol Lysis Reagent (Qiagen, Venlo) according to the manufacturer’s instructions. RNA quality and quantity were determined using a Nanodrop spectrophotometer ND-1000 (Thermo Scientific, Rockland) while the RNA integrity was verified using the Qiaxcel qiacube (Qiagen, Venlo). Isolated intact and high quality RNA was further processed for microarray analysis.

#### cRNA labelling and hybridization

Total RNA was linearly amplified and labelled using the Low Input Quick Amplification Labelling Kit (LIQA, Agilent Technologies, Santa Clara, CA, USA) according to the manufacturer’s instructions. In brief, 100 ng RNA was reverse transcribed into cDNA using oligo dT primers. Subsequently, cDNA was converted into cRNA and amplified and the resulting cRNA of each sample was labelled once with Cy3-CTP and once with Cy5-CTP. The RNeasy mini spin column kit (Qiagen, Venlo) was then used to purify the cRNA samples. cRNA yield, quality and dye incorporation efficiency were verified using a Nanodrop spectrophotometer.

We used Agilent’s Zebrafish Gene Expression Microarray V3 in a 4x44k format, which is a full genome microarray developed by Agilent and containing 43,803 *Danio rerio* probes. 825 ng Cy3 and 825 ng Cy5 labelled and purified cRNA were hybridized onto these microarrays for 17 h at 65°C in a rotating (10 rpm) hybridization oven (Agilent Technologies). Four biological replicates of each condition were used and an A-optimal design was used as the hybridization design for each body region separately [19].

After hybridization, slides were immersed in Agilent wash buffers, acetonitrile and in stabilization and drying solution (Agilent Technologies) to wash and protect against ozone-induced Cy5-degradation. Microarray slides were scanned using a Genepix 4100A confocal scanner (Axon Instruments, Union City, CA, USA) at a resolution of 5 μm and excitation wavelengths of 635 nm and 532 nm in an ozone-free environment (NoZone scanner enclosure, SciGene, Sunnyvale, CA, USA). Images were analyzed for spot identification and for quantification of the fluorescent signal intensities using the Genepix Pro software 6.1 (Axon Instruments).

#### Statistical processing of raw microarray data

Statistical analyses of raw microarray data were performed using the R package Limma [20] as described by Vergauwen et al. [21] for each body region separately. Both knockdown conditions were contrasted against SCMO. Spots for which the criterion FG < BG + 2SD (FG: foreground, BG: background, SD: standard deviation of the local background of the entire array [22]) was true for all arrays in the dataset, were excluded from analysis. Background correction was carried out using a normal-exponential convolution model [23]. Within-array adjustment was done by Loess normalization [24], which performs an intensity-dependent normalization of the ratio of red and green. Linear models were fitted to intensity ratios, after which probes were ranked in order of evidence of differential transcription using an empirical Bayes method [20]. Contrasts were fitted to the linear models and cut off at FDR < 0.05 (false discovery rate, multiple testing correction using the Benjamini-Hochberg procedure) and |log2FC| > 0.585 (log2 fold change, corresponding to a fold induction of at least 1.5 or -1.5). Raw and analysed microarray data have been deposited in NCBI’s Gene Expression Omnibus (GEO, http://www.ncbi.nlm.nih.gov/geo) and are accessible through the GEO series accession number GSE61625 [reviewer only link: http://www.ncbi.nlm.nih.gov/geo/query/acc.cgi?token=ejojsqegzbmfnaf&acc=GSE61625].

#### Biological interpretation of microarray data

Before further data analysis, for distinct probes on the array targeting identical transcripts with identical names, Agilent primary accession numbers and Genbank accession numbers, the mean of the log_2_FC values was calculated. Hierarchical clustering analysis of the samples was performed using MultiExperiment Viewer software 4.8.1 (MeV, http://www.tm4.org/mev.html) based on Pearson correlation with average linkage with bootstrapping. The biological function of significantly differentially expressed transcripts in D1D2MO and D3MO was analyzed in Blast2GO (www.blast2go.org). The most prominently affected biological processes were identified using pie charts of the blast2GO score which takes into account the number of affected transcripts within each gene ontology (GO) class and the level of specificity of the respective GO class. This score was cut off at 5 to 10% of the total number of annotated transcripts in their respective condition. Subsequently, these GO classes were grouped manually into broader biological categories (e.g. metabolic process, development) to create a general overview of the data (See also S1 Table). Additionally, KEGG pathway analysis was used to identify strongly affected pathways. Only pathways containing at least 3 differentially expressed enzymes were considered (See also S1 Table).

### 4. Analysis of the knockdown phenotype

#### Assessment of morphological effects

An early life stage (ELS) test was performed in three experimental replicates for each condition, resulting in a total sample size of 120 embryos per knockdown condition. The test was performed in 48-well plates with one embryo per well. The first replicate contained 24 embryos per knockdown condition. The second and third replicates each contained 48 embryos per knockdown condition. For the morphological analysis we also included an uninjected control (UC) group and the assessing researcher was blinded with respect to the knockdown condition of the embryos. From 24 hpf up to 96 hpf, lethal and sublethal morphological and physiological endpoints were evaluated every 24 h using a stereomicroscope. The observed endpoints per time point are listed in Table 1. The heart rate of the embryos was recorded at 24 hpf and at 48 hpf by counting the heartbeats for 10 seconds (n = 96, from two experimental replicates). For morphological analysis and recording digital images at 96 hpf, embryos were lightly anaesthetized in 0.1 g/L (pH 7.3 ± 0.1) MS-222 (Tricaine, Sigma-Aldrich, Cas nr: 886-86-2). To determine the effects on growth, the length of larvae along the body axis from head to the tail tip was measured at 96 hpf on digital images (acquired using a Canon EOS 600D, 18 Mpix) using the Image J software (version 1.47).

**Table 1:** Observed endpoints at specific time points during the zebrafish early life stage test.

	Developmental time point (hpf)				
**Observed endpoint**	24	48	54	72	96
Coagulation	**•**	**•**	**•**	**•**	**•**
Tail detachment	**•**				
Somite formation	**•**				
Presence of heart beat	**•**	**•**	**•**	**•**	**•**
Hatching		**•**	*	*	**•**
Eye underdevelopment^¶^	**•**	**•**			
Pigmentation	**•**	**•**		**•**	**•**
Oedema pericard	**•**	**•**		**•**	*
Oedema yolk	**•**	**•**		**•**	**•**
Oedema head	**•**	**•**		**•**	**•**
Heart rate^†^	*	**•**			
Malformation head	**•**	**•**		**•**	**•**
Malformation eye	**•**	**•**		**•**	**•**
Malformation mouth				**•**	**•**
Malformation otolith/sacculi	**•**	**•**		**•**	**•**
Malformation heart	**•**	**•**		**•**	**•**
Malformation pectoral fin					**•**
Malformation yolk	**•**	**•**		**•**	**•**
Malformation/irregular somites	**•**	**•**		**•**	**•**
Malformation tail	**•**	**•**		**•**	**•**
Curve in tail				**•**	**•**
Swim bladder inflation					*
Blood accumulation	**•**	**•**		**•**	**•**
Length^†^	** **	** **	** **	** **	*

Except for continuous variables, all endpoints were visually evaluated for presence or absence using a binary scoring system.

**•** Indicates at which time points each endpoint was observed.

* Indicates which endpoints were significantly affected by at least one of the knockdown conditions when compared to SCMO.

† Continuous variable

¶ Recognized by normal eye shape resembling younger embryos and/or by less iridiphores at certain timepoint.`

Hpf = hours post fertilization.

#### Assessment of larval motility and energy stores

Based on the transcriptional patterns indicating effects on muscle function and energy metabolism, we performed additional analyses of motility and energy stores. For analysis of motility SCMO, D1D2MO and D3MO embryos were transferred to individual wells of a 48-well plate and were incubated up to 96 hpf. In each plate, embryos from each condition were included and the total sample size for each condition was 24. At 96 hpf, larval movements were recorded and monitored with a Zebrabox (ViewPoint, Lyon, France) for 75 minutes in a 100% full light period (1160.7 lux), using the video tracking mode with the detection threshold set at 126. The first 30 minutes were used as an acclimation period and were therefore not included in the analysis. The total distance (mm) travelled during the 45 remaining minutes was calculated.

For measurement of energy stores, SCMO, D1D2MO and D3MO embryos were raised in Petri dishes and incubated up to 72 hpf. Four biological replicates of each condition, each replicate containing 48 pooled 72 hpf-old whole larvae, were collected. Carbohydrates, lipids and proteins were extracted and measured as described earlier [21]. In brief, larvae homogenized in deionized water were used for total lipid analysis. Total lipid extraction was performed based on the chloroform-methanol protocol according to Bligh and Dyer [25] and absorption was measured at 375 nm, relative to a calibration curve of cholesterol (Acros Organics, Geel, Belgium) in chloroform. From a separate homogenate, proteins were precipitated using perchloric acid and the total protein content was determined using the Bradford method [26] by measuring absorption at 592 nm, relative to a calibration curve of bovine serum albumin (Sigma, Bornem, Belgium) in water. From the same larval homogenate, the total carbohydrate content was measured in the supernatant, using Anthrone reagent prepared according to Roe and Dailey [27] and absorption was measured at 630 nm, relative to a calibration curve of glycogen (Sigma, Bornem, Belgium) in perchloric acid. Total caloric content was calculated using the appropriate energy combustion values (39.5 kJ/g lipid, 24 kJ/g protein and 17.5 kJ/g carbohydrates [28]).

#### Statistical analysis of morphological and physiological data

*Statistical tests were performed using IBM SPSS statistics (version 20) and Graphpad Prism (version 6.0). A p-value below 0.05 was considered significantly different. One-way analysis of variance (ANOVA) with Bonferroni corrected post hoc tests was used to determine significant effects between the MO groups in larval length and heart rate. A Kaplan-Meier survival curve analysis was conducted with log-rank Mantel-Cox test to analyze the hatching results in a time-dependent manner. A chi square test followed by pairwise chi square tests with Bonferroni correction was performed to identify significant differences in frequencies of malformations such as non-inflated swim bladder and pericardial oedema among all conditions. Kruskal-Wallis tests followed by Bonferroni corrected Mann-Whitney U tests were performed to determine significant differences in the travelled distance, the carbohydrate, protein and lipid contents, and the total caloric content. Knockdowns were initially compared to SCMO. If further differences were observed, additional comparisons were made.

## Results

### 1. mRNA expression signature of D1D2MO and D3MO in head, abdomen and tail regions

**Fig 1:**
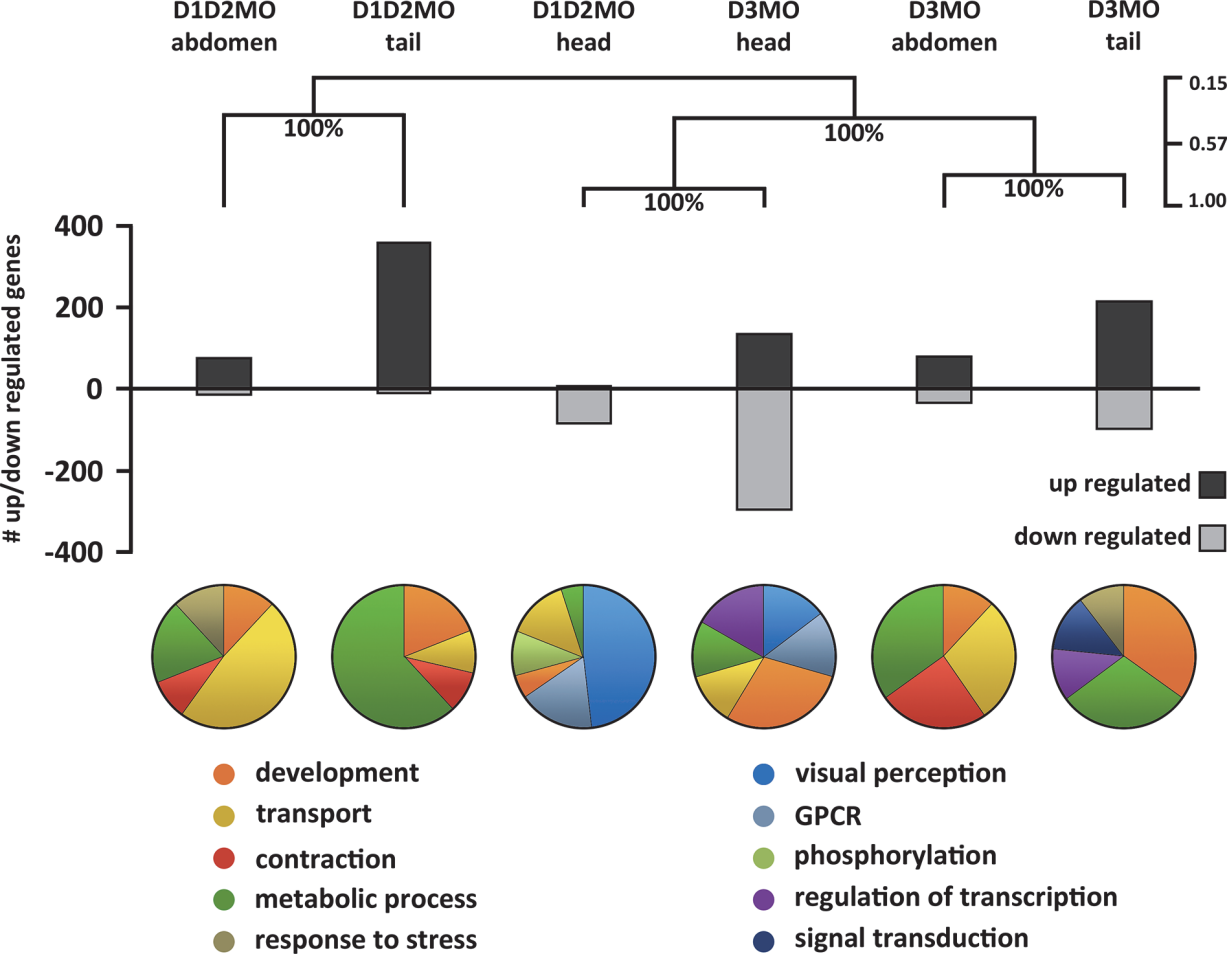
mRNA expression signature in head, abdomen and tail of deiodinase knockdown larvae at 72 hpf. The results of unsupervised hierarchical clustering (Pearson correlation, average linkage) generated on the total set of significantly differentially expressed transcripts in D1D2MO and D3MO in the head (H), abdomen (A) and tail (T) regions are shown at the top. This analysis shows (dis)similarities among expression patterns in the different body parts of the deiodinase knockdowns. The transcriptional response in the head was similar in both deiodinase knockdowns, and distinct from that in other body parts. In contrast, the response in abdomen and tail was knockdown-specific but similar among these two body parts. Bootstrap support values (in %) are denoted at each node, showing the level of confidence in each step of the clustering. The legend on the right shows the Pearson correlation coefficient. The bar chart gives an overview of the number of up- and downregulated transcripts for each knockdown in each body part. Pie charts summarize the most important GO classes (gene ontology: biological process) affected by each knockdown in each body part. GO classes were manually grouped into broad biological categories. GO classes received a score calculated in Blast2GO based on the number of sequences they contained and the level of their specificity. GPCR = G-protein coupled receptor signalling pathway. D1D2MO: combination of deiodinase type 1 and type 2 morpholino-injected, D3MO: deiodinase type 3 morpholino-injected, and SCMO: standard control morpholino-injected.

1056 unique transcripts were differentially expressed in at least one of the studied conditions and/or body regions. Hierarchical clustering analysis across these responding transcripts revealed that the transcriptional response in the head was similar in both deiodinase knockdowns, although the response of D3MO was stronger (Fig 1). Most of these differentially expressed transcripts in the head were downregulated. In contrast, the response in abdomen and tail was knockdown-specific and similar among these two body parts, and transcripts were mainly upregulated. An overview of the numbers of significantly up- and downregulated mRNAs for D1D2MO and D3MO in each body part is also given in Fig 1. In the head, a higher number of differentially expressed transcripts was observed in D3MO (293) in comparison to D1D2MO (82). In the tail, the number of differentially expressed transcripts in D1D2MO (362) and D3MO (311) was similar. In the abdomen, the number of significantly differentially expressed transcripts was limited and similar in both D1D2MO (88) and D3MO (112).

**Fig 2:**
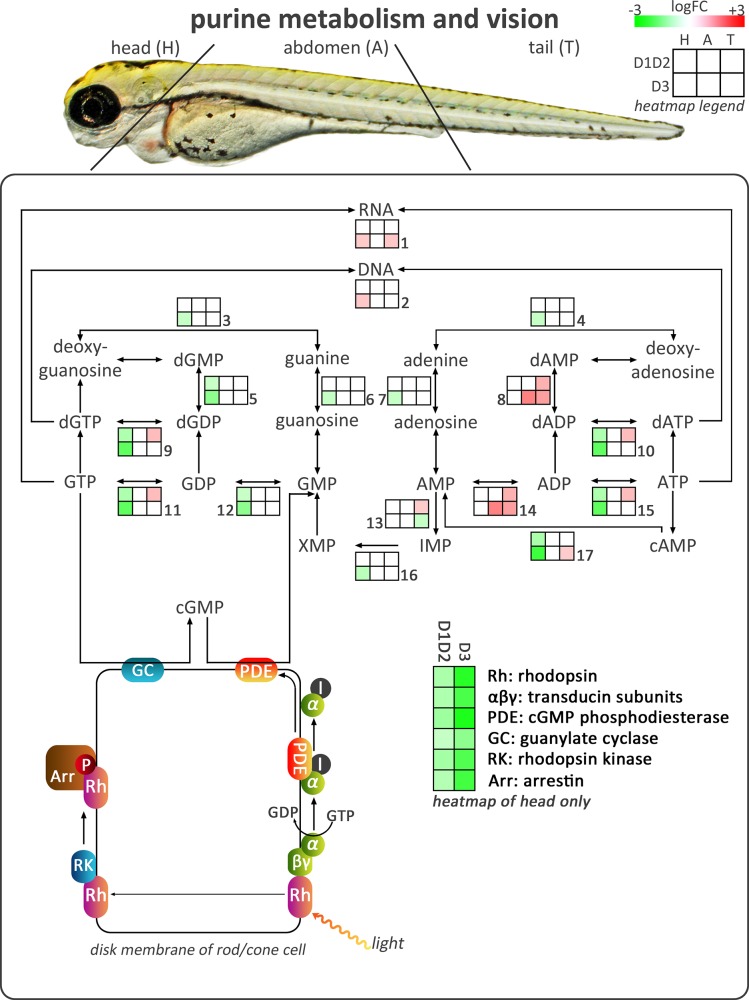
Overview of transcriptional effects on purine metabolism and vision. The figure shows the affected part of the KEGG pathway ‘purine metabolism’, and its link to vision. Small heat maps show logFC values of differentially expressed transcripts coding for the enzymes involved in the pathway. Green indicates downregulated (Log2FC ≤ -0.585, FDR ≤ 0.05), and red indicates upregulated (Log2FC ≥ 0.585, FDR ≤ 0.05) transcripts. White boxes indicate that transcripts were not differentially expressed. D1D2: combination of deiodinase type 1 and type 2 morpholino-injected, D3: deiodinase type 3 morpholino-injected, I: inhibitory subunit of PDE. Numbers refer to S2 Table listing the details of the differentially expressed transcripts.

To functionally characterize the altered mRNA expression signatures in the different body parts of D1D2MO and D3MO in general, the most important GO classes were identified using a score based on both the number of sequences and the specificity of the GO class. Fig 1 shows pie charts of broader biological categories into which these GO classes were grouped to provide an overview of the most important processes affected by each knockdown in each body region. (See S1 Table for a detailed overview of the GO analysis). In the head of D1D2MO and D3MO there was an important effect on visual perception and G-protein coupled receptor signalling pathway, which are both involved in vision. Additionally, we identified purine metabolism as the most strongly affected KEGG pathway in the head (See S1 Table for a detailed overview of the KEGG analysis). Fig 2 shows the most important enzymes of the purine metabolism pathway linked to the phototransduction pathway implicated in vision. From this figure, it is clear that D1D2MO and D3MO for the most part downregulated the same transcripts, but that D3MO furthermore downregulated additional transcripts.

**Fig 3:**
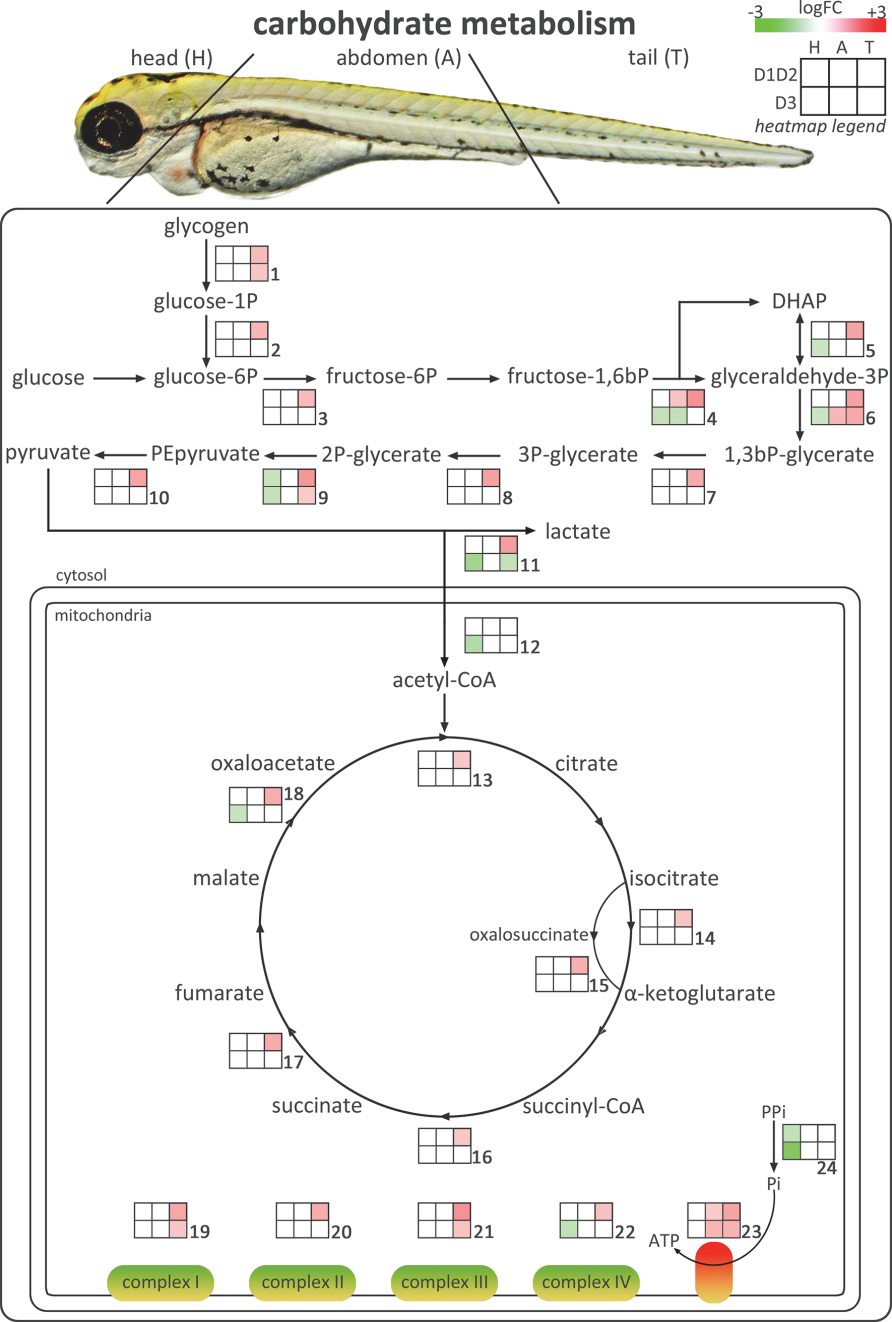
Overview of transcriptional effects on carbohydrate metabolism. The figure shows carbohydrate metabolism starting with glycogen breakdown, and including glycolysis/gluconeogenesis, the citric acid cycle and oxidative phosphorylation. Small heat maps show logFC values of differentially expressed transcripts coding for the enzymes involved in the pathway. Green indicates downregulated (Log2FC ≤ -0.585, FDR ≤ 0.05), and red indicates upregulated (Log2FC ≥ 0.585, FDR ≤ 0.05) transcripts. White boxes indicate that transcripts were not differentially expressed. D1D2: combination of deiodinase type 1 and type 2 morpholino-injected, D3: deiodinase type 3 morpholino-injected. Numbers refer to S2 Table listing the details of the differentially expressed transcripts.

**Fig 4:**
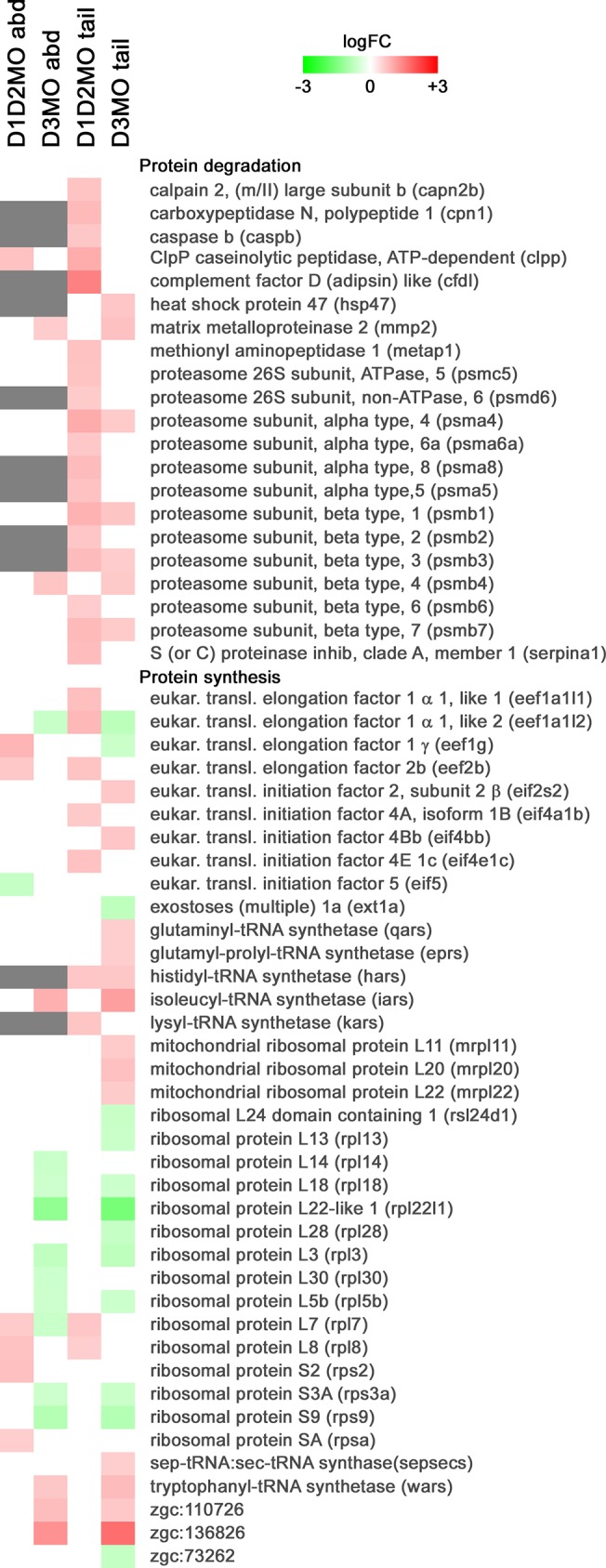
Heat map of differentially expressed transcripts involved in protein metabolism in abdomen and tail region. Green indicates downregulated (Log2FC ≤ -0.585, FDR ≤ 0.05), and red indicates upregulated (Log2FC ≥ 0.585, FDR ≤ 0.05) transcripts. White boxes indicate that transcripts were not differentially expressed. Grey boxes indicate that transcripts were removed from analysis in a specific body part because FG < BG + 2SD (See Materials and Methods). Abd: abdomen, D1D2MO: combination of deiodinase type 1 and type 2 morpholino-injected, D3MO: deiodinase type 3 morpholino-injected. All differentially expressed transcripts are listed with their details in S2 Table.

**Fig 5:**
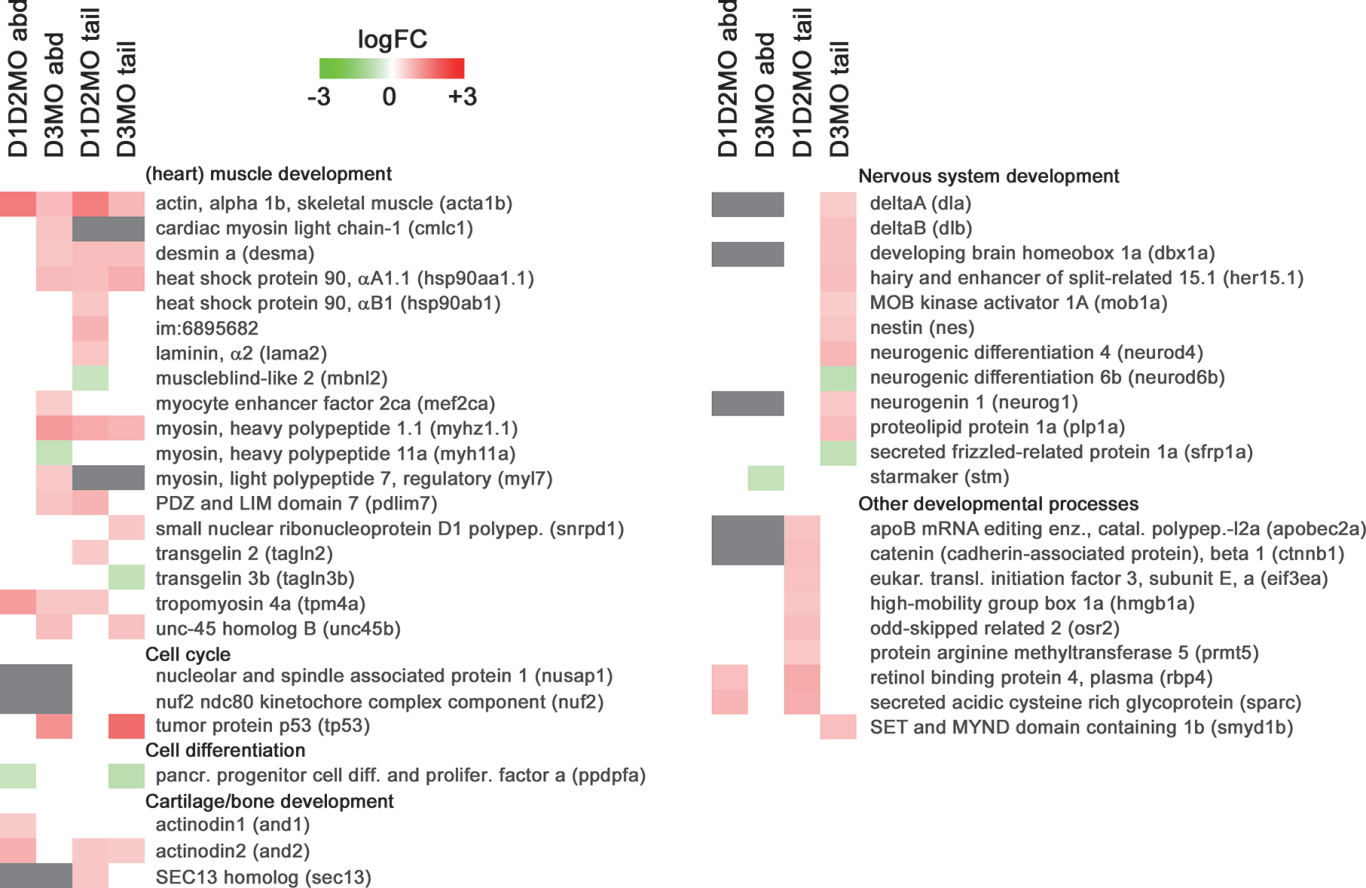
Heat map of differentially expressed transcripts involved in developmental processes in abdomen and tail region. Green indicates downregulated (Log2FC ≤ -0.585, FDR ≤ 0.05), and red indicates upregulated (Log2FC ≥ 0.585, FDR ≤ 0.05) transcripts. White boxes indicate that transcripts were not differentially expressed. Grey boxes indicate that transcripts were removed from analysis in a specific body part because FG < BG + 2SD (See Materials and Methods). Abd: abdomen, D1D2MO: combination of deiodinase type 1 and type 2 morpholino-injected, D3MO: deiodinase type 3 morpholino-injected. All differentially expressed transcripts are listed with their details in S2 Table.

In abdomen and tail of both knockdown conditions, the GO classes representing the differentially expressed transcripts were mainly involved in metabolic processes, development and contraction, together with some more general processes such as transport, signal transduction, regulation of transcription and response to stress. Especially in the tail of D1D2MO a lot of transcripts involved in carbohydrate metabolism, including gluconeogenesis/glycolysis, the citric acid cycle, and the electron transport chain, were upregulated, while this was not the case in D3MO. These pathways were also identified in the KEGG pathway analysis and are shown in Fig 3. Additionally, transcripts involved in protein degradation were upregulated in D1D2MO tail. In D3MO tail, differentially expressed transcripts were mainly involved in protein turnover and part of this response was also observed in D3MO abdomen. From the heat map (Fig 4), it is clear that the response in abdomen was limited. Fig 5 shows a heat map of the differentially expressed transcripts included in those GO classes that were grouped into the broad class of development. In the tail of both knockdown groups, several transcripts coding for proteins involved in muscle development were upregulated. In abdomen of D3MO in particular, several transcripts involved in muscle development and contraction, including some specifically involved in heart development (e.g. cardiac myosin light chain-1), were differentially transcribed (mostly upregulated). Furthermore, in the tail of D3MO we specifically observed altered transcriptional expression of genes involved in nervous system development.

### 2. Analysis of the knockdown phenotype

**Fig 6:**
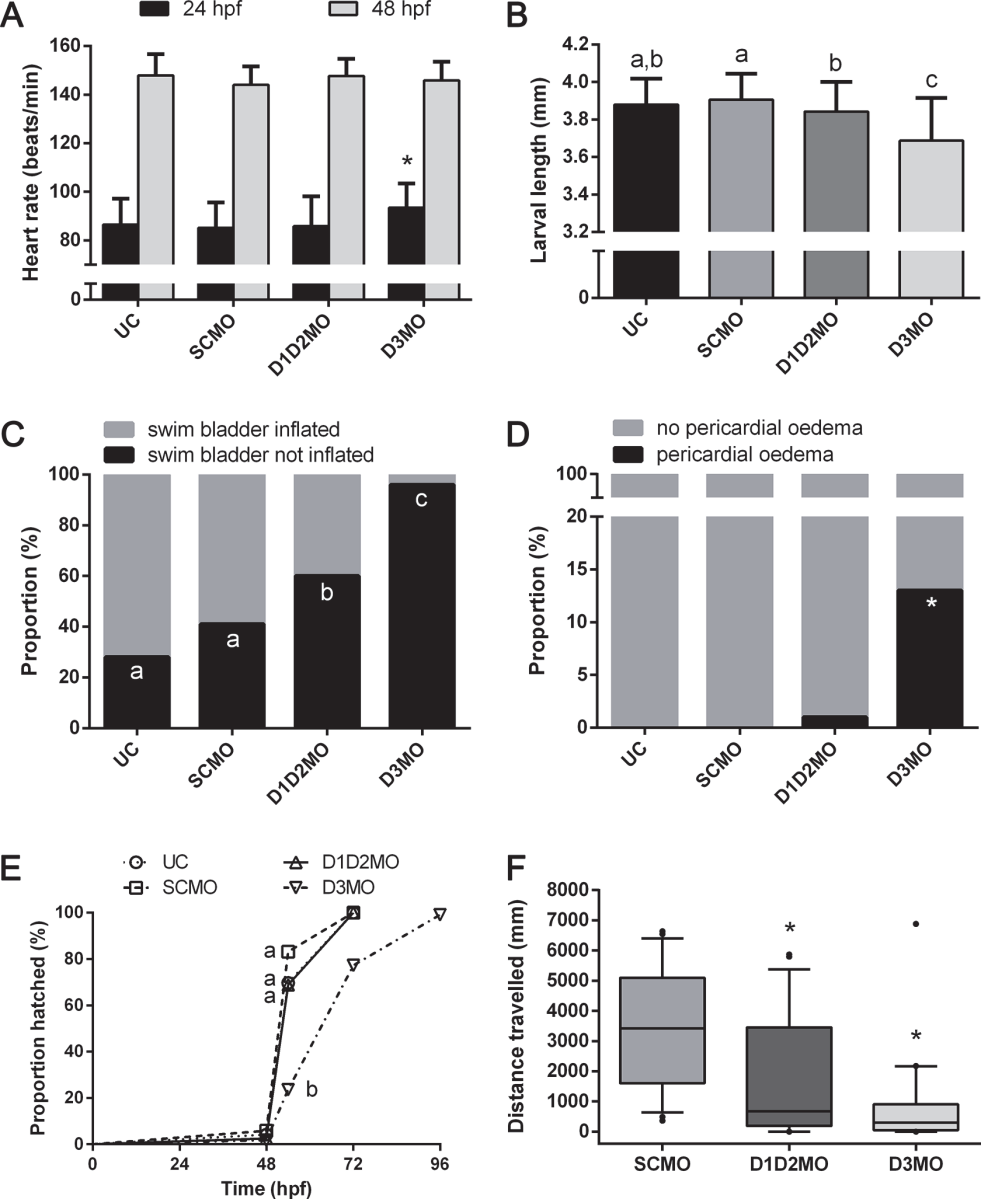
Phenotypic effects observed in D1D2MO and D3MO zebrafish larvae. (A) heart rate at 24 and 48 hpf, (B) larval length at 96 hpf, (C) swim bladder inflation success at 96 hpf, (D) frequency of pericardial oedema at 96 hpf, (E) hatching success as a function of time, and (F) larval motility at 96hpf: boxplot with median, 10 and 90 percentile of the travelled distance during 45 minutes. In (A) and (B), data are shown as average ± SD and SD was calculated over all individual embryos. Knockdowns were initially compared to SCMO, and * indicates a significant difference from SCMO. If further differences were observed, additional comparisons were made, and different letters indicate significant differences among experimental groups. In (E), letters indicate significant differences among hatching curves taking all time points into account. D1D2MO: combination of deiodinase type 1 and type 2 morpholino-injected, D3MO: deiodinase type 3 morpholino-injected, UC: uninjected control. SCMO: standard control morpholino-injected.

All endpoints evaluated in the ELS test, are listed in Table 1. Those endpoints showing significant effects are shown in Fig 6A–6F. Other endpoints were not significantly affected by the knockdown conditions. At 24 hpf, the average heart rate was significantly increased in D3MO compared to SCMO and D1D2MO embryos (Fig 6A). At 48 hpf, the average heart rate in D3MO was similar to control levels. Compared to SCMO, the average body length at 96 hpf was significantly reduced in D1D2MO and D3MO by 0.07 mm and 0.22 mm respectively, the reduction being significantly stronger in D3MO (Fig 6B). At 96 hpf, swim bladder inflation was observed in 59% (70 of 119 larvae) and 72% (86 of 119 larvae) of the injected (SCMO) and uninjected control (UC) embryos respectively. Swim bladder inflation was significantly impaired in D1D2MO (40% inflated, 48 of 119 larvae) and D3MO (4% inflated, 4 of 110 larvae) compared to the controls, and the effect was significantly stronger in D3MO (Fig 6C). At 96 hpf, 13% (14 of 110 larvae) of D3MO embryos had developed pericardial oedema in contrast to only 1% (1 of 119 larvae) of D1D2MO and 0% (0 of 119 larvae) of SCMO (Fig 6D). D3MO significantly delayed hatching (Fig 6E), but at 96 hpf all D3MO embryos had hatched.

**Fig 7:**
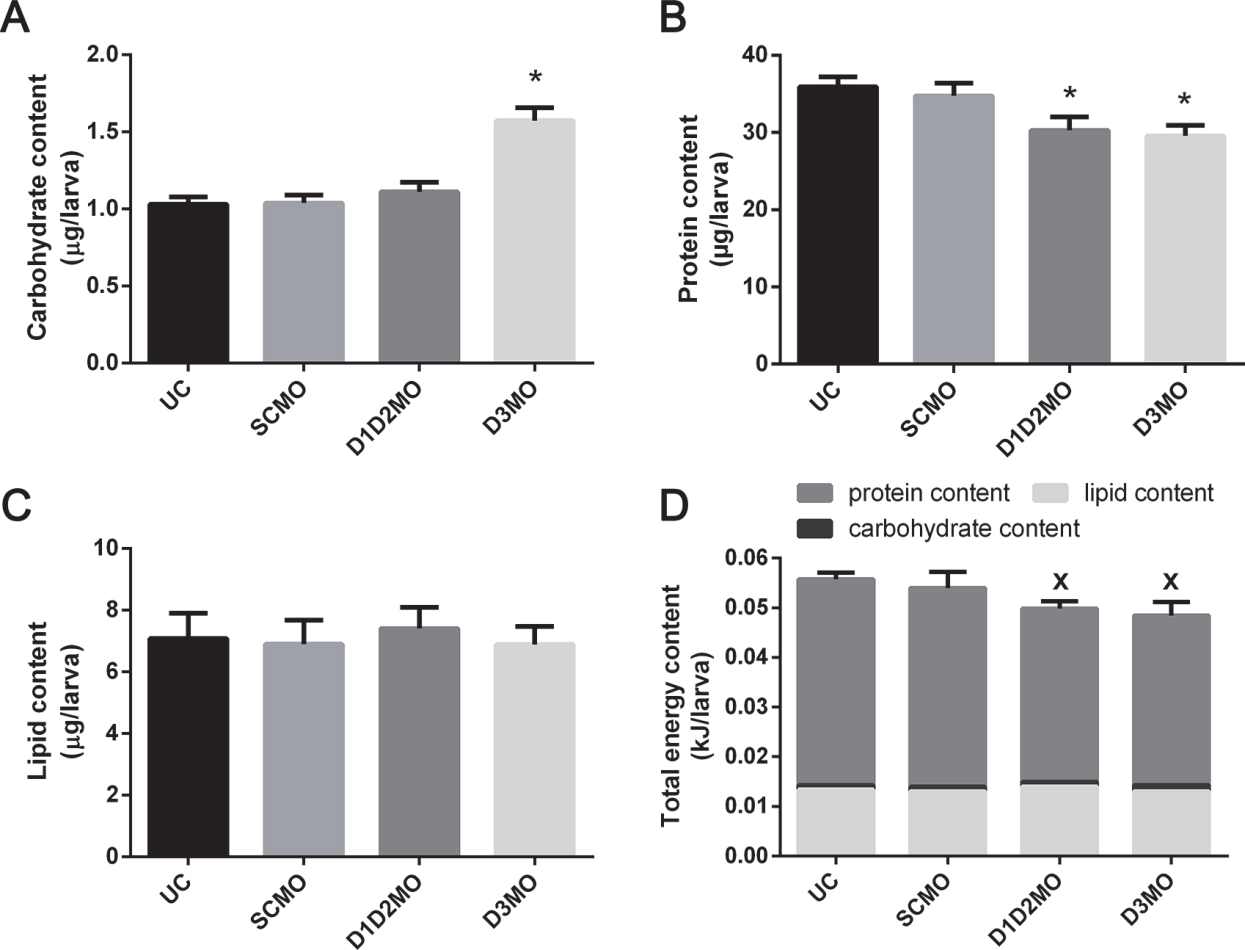
Effect of deiodinase knockdown on energy status of zebrafish larvae. (A) carbohydrate content, (B) protein content, (C) lipid content, and (D) total energy content in zebrafish larvae at 72 hpf. Data are shown as average ± SD and SD was calculated over four biological replicate samples. Error bars in (D) show SD of total energy content. * indicates a significant difference from SCMO. X indicates a significant difference from the total energy content of UC. D1D2MO: combination of deiodinase type 1 and type 2 morpholino-injected, D3MO: deiodinase type 3 morpholino-injected, UC: uninjected control. SCMO: standard control morpholino-injected.

Larval motility at 96 hpf showed that the distance travelled was significantly reduced compared to SCMO in both D1D2MO and D3MO (Fig 6F). At 72 hpf, the carbohydrate content in D3MO was significantly increased compared to SCMO (Fig 7A). In both D1D2MO and D3MO the protein content was significantly decreased compared to SCMO (Fig 7B). Lipid levels were unaffected (Fig 7C). Although in D1D2MO and D3MO the total energy content was decreased by 11% (0.005860611 kjoule of 0.055707 kjoule) and 13% (0.007307004 kjoule of 0.055707 kjoule) compared to UC, the total energy content was not significantly different from that of SCMO (Fig 7D).

## Discussion

Although it is established that THs have a fundamental role in development, many uncertainties remain regarding the role of TH activating and inactivating deiodinases during early vertebrate development. In deiodinase knockdown zebrafish, we investigated both mRNA expression profiles and phenotypic effects. Phenotypic effects were less severe or sometimes absent in D1D2MO compared to D3MO (Fig 6). Based on the transcriptional expression profiles, both knockdown conditions caused a highly similar response in the head while the responses in abdomen and tail differed between the two knockdowns (Fig 1). Many of these responses would probably have remained undetected if transcriptional profiling were performed on whole organisms. The response in the abdomen was limited, possibly because the tissue type composition of this region was still too complex. Here we discuss the specific responses in the different body parts in relation to phenotypic effects.

### 1. D1D2MO and especially D3MO decrease expression of transcripts involved in purine metabolism and vision

The GO and KEGG analysis showed that the most prominently affected processes in the head were purine metabolism and vision (Fig 2). In a human disease context, a significant correlation between thyroid function and purine nucleotide metabolism has been established [29]. While hypothyroidism has been linked to hyperuricemia (increased levels of urate, a breakdown product of purine metabolism) due to decreased renal excretion [30]), the observed association between hyperthyroidism and hyperuricemia was suggested to be due to increased purine turnover [29]. Although the transcriptional changes in the current study clearly showed that purine metabolism was affected by deiodinase knockdown in developing zebrafish larvae, the prominent downregulation in D3MO contradicts the hypothesized increased turnover. It is however remarkable that this is accompanied by downregulation of transcripts involved in phototransduction. Phototransduction is an important G protein signalling pathway in which the photoreceptor-specific G protein, transducin, activates cGMP phosphodiesterase, necessary for transferring the signal to the brain. Due to the importance of cGMP levels in this process, purine metabolism and vision are tightly linked. All major components of the phototransduction pathway were downregulated in both knockdowns, including rhodopsin, transducin and phosphodiesterase involved in the excitation phase, and guanylate cyclase, rhodopsin kinase, and arrestin involved in recovery or adaptation. Whole mount *in situ* hybridization experiments have shown localized expression of both D2 and D3 in the retina of developing zebrafish embryos [13,31,32]. Together with our previous observations of decreased eye size in D3MO zebrafish at 29 and 96 hpf [10], this suggests that eye development and function are affected by deiodinase knockdown. Eye development has also been shown to be regulated by THs in several other vertebrates including rodents, amphibians and chicken [33]. For example, Sevilla-Romero et al. [34] discovered that the retina from hypothyroid rat pups had fewer dividing progenitor cells and impaired photoreceptors, as well as an overall reduced thickness. In the current study, we did not observe eye underdevelopment or eye malformations by external visual observation under a stereomicroscope (Table 1). However, this does not exclude the possibility of more subtle effects that could be observed using image acquisition and more refined analysis techniques. Overall, these findings suggest that a fine-tuned TH balance is essential to coordinate vertebrate eye development. Disruption of rhodopsin expression in mice was shown to reduce the sensitivity to light [35]. We hypothesize that both D1D2 and D3 deficiency impair eye development and function, reduce light sensitivity and disrupt rhodopsin recovery following bleaching. The observed transcriptional expression response in D3MO was stronger. Therefore, effects on light perception may also be stronger.

### 2. Deiodinase knockdown affects carbohydrate and protein metabolism and muscle development in the tail

In the tail both D1D2MO and D3MO influenced transcriptional expression of transcripts involved mainly in metabolism and development and both conditions were characterized by pronounced upregulation of transcripts (Fig 1). However, the clustering indicates an important difference between the responses to either knockdown, which is obvious in Figs 3–5.

#### D1D2MO and D3MO have different effects on carbohydrate and protein metabolism

While the transcriptional response related to metabolism in D3MO was mostly limited to protein turnover (Fig 4), the response of D1D2MO was characterized by consistent upregulation of a remarkable number of transcripts coding for key proteins in carbohydrate metabolism, including glycogen breakdown, almost all steps of gluconeogenesis/glycolysis, the citric acid cycle and all five complexes of the electron transport chain (Fig 3). High TH levels are known to cause increased mitochondrial respiratory function, increased oxygen consumption, increased basal metabolic rate, and weight loss [36]. The consistent upregulation in D1D2MO tail could reflect a compensatory response to a decreased metabolic rate due to low T_3_ levels. The liver is the central organ responsible for regulating carbohydrate metabolism and so far localized expression of D1 has been shown in liver of 48, 72, 96 and 120 hpf zebrafish embryos [17,31,37]. Although changes in energy metabolism in the tail are mainly attributed to muscle tissue, they are possibly indirectly affected by the liver.

In D3MO larvae, the transcriptional response involved in carbohydrate metabolism was limited, but carbohydrate stores were increased. The impact of TH on glucose metabolism has been studied extensively in humans and rats, where T_3_ is known to stimulate gluconeogenesis and cause hyperglycemia [38–40]. Among other effects, T_3_ stimulates hepatic glucose production and increases GLUT4-mediated transport of glucose into skeletal muscle and adipose cells [41–43]. This could explain the increased carbohydrate levels in D3MO zebrafish larvae. Although this is often related to increased expression of enzymes involved in gluconeogenesis [44], this was not observed in our study. This gluconeogenesis stimulating effect has also been attributed to an excessive availability of alanine as a substrate for gluconeogenesis, resulting from a thyrotoxicosis-associated catabolic state in isolated rat livers [45]. Correspondingly, we found decreased protein levels in D3MO larvae (Fig 7B). Increased proteolysis in skeletal muscle of hyperthyroid rats was found to be mediated mainly via the ubiquitin-proteasome pathway [46]. Among the transcriptional regulations involved in metabolism in D3MO, we mostly found transcripts involved in protein turnover including both protein degradation (proteasome subunits) and protein synthesis (Fig 4). Such conversion of proteins to carbohydrates could be partly responsible for the observed reduced growth of D3MO (Fig 6B), because structural proteins play an important part in growth (e.g. muscle) and because such conversions are energy expensive. Additionally, hyperthyroidism has been related to a decrease in metabolic efficiency by controlling the coupling of mitochondrial oxidative phosphorylation and the cycling of extramitochondrial substrate/futile cycles [47,48]. In contrast to free-feeding organisms, zebrafish embryos depend on a limited energy resource in the yolk up until the start of free-feeding around 5 days post fertilization. Therefore, the larvae analysed in this study could not compensate for a potentially increased energy loss. Although a potential increase of the metabolic rate and decrease of metabolic efficiency in D3MO did not result in a significant decrease of total available energy compared to SCMO, it could have contributed to the reduced growth. In contrast, in the case of D1D2MO, hypothyroidism is known to be related to reduced gluconeogenesis and resulting episodes of hypoglycemia [49]. Therefore, the observed upregulation of almost all enzymes involved in gluconeogenesis in D1D2MO larvae could have been a compensating mechanism facilitating maintenance of normal carbohydrate levels in D1D2MO larvae. D1D2MO also showed many upregulated transcripts involved in protein degradation and concurrently protein levels were lower than in SCMO. This could be related to decreased growth. Methimazole, an inhibitor of TH synthesis, has also been shown to cause growth retardation in 5-day-old zebrafish larvae [50] and growth arrest in 3-week-old zebrafish larvae [16]. These effects could be due to the absence of the TH stimulus needed to drive growth and development. We conclude that, even though both knockdowns resulted in decreased growth, the transcriptional pattern indicates that this was caused by different mechanisms.

TH is also known to stimulate both lipolysis and lipogenesis [51]. Since lipid levels were similar to control levels in both knockdown conditions (Fig 7C), and total energy stores were not significantly reduced compared to SCMO (Fig 7D), we conclude that altered triglyceride cycling was not an important contributor to the observed metabolic state.

#### Both knockdowns affect muscle development and motility

TH is known to be involved in muscle development and muscle maturation, which is associated with a switch between myosin isoforms during postnatal development in mammals [52–54] and during metamorphosis in tadpoles [55]. In the tail of both deiodinase knockdown conditions, several transcripts coding for proteins involved in muscle development were upregulated (Fig 4). These genes included *hsp90* and *unc-45*, involved in sarcomere formation during muscle development; *mef2ca*, a transcription factor regulating muscle gene expression; tropomyosin, myosin heavy polypeptide and myosin regulatory light polypeptide which are sarcomere components and desmin, which connects the sarcomere to the cytoskeleton.

We also observed decreased motility of both knockdowns (Fig 6F). Since total energy stores were not significantly decreased compared to SCMO, factors other than energy availability could be related to the observed decreased motility. We did not observe an increased incidence of malformations of the pectoral fins, malformations or irregularities of somites, nor of malformations or curvatures of the tail (Table 1). A first important contributor could be impaired muscle development, indicated by the differentially expressed transcripts involved in muscle development together with the decreased protein levels. A second factor could be impaired visual perception, for which we found evidence at the transcriptional level (See 4.1). A third factor could be impaired nervous system development, which has been linked to maternal thyroid hormone contribution in zebrafish [56]. We observed transcriptional alterations related to nervous system development in D3MO but not in D1D2MO larvae (Fig 5). Motility is needed for hatching and subsequent emergence to the water surface to inflate the swim bladder [57]. We also observed impaired swim bladder inflation in both knockdowns and delayed hatching in D3MO (Fig 6C and 6E), but we must note that it is difficult to distinguish between cause and effect in this context. For example, Liu and Chan [50] reported that T_4_-supplemented larvae showed less compact myotome arrangement and that they showed no free-swimming behaviour. The authors suggested that this was due to the defective swim bladder. (See 4.3 for more detailed discussion of swim bladder inflation)

### 3. Deiodinase knockdown affects energy metabolism, muscle contraction, heart function and swim bladder inflation in abdomen

In abdomen, only few transcripts were differentially expressed, probably because of the high remaining tissue diversity in this body part (Fig 1). Most of these transcripts were involved in metabolism and (heart) muscle development and contraction. Especially in the abdomen of D3MO larvae, transcripts involved in energy metabolism were differentially regulated. These transcripts were mostly limited to protein synthesis and showed a response highly similar to the one in D3MO tail (Fig 4), which leads us to suggest that it is due to increased muscle protein turnover both in abdomen and tail (See 4.2).

#### D3MO affects heart function

TH balance is known to be essential for heart morphology and function, and T_3_ has previously been shown to increase heart rate [58,59]. Furthermore, shortening of the action potential and the resulting tachycardia increases susceptibility to heart failure, known as thyrotoxic heart and is thought to be related to sympathetic adrenalin signalling [58,60]. We observed an increased heart rate at 24 hpf in D3MO embryos, while at 48 hpf the heart rate was comparable to that of the controls (Fig 6A). At 72 hpf, several transcripts involved in muscle development and contraction, including some specifically involved in heart development (e.g. cardiac myosin light chain-1), were differentially transcribed (mostly upregulated) in abdomen of D3MO in particular (Fig 5). Moreover, at 96 hpf we observed an increased occurrence of pericardial oedema, possibly a sign of heart failure (Fig 6D). Contrastingly, D1D2MO heart rate was comparable to controls combined with only little transcriptional response and no pericardial oedema.

#### Both knockdowns impair swim bladder inflation

T_3_ is known to drive growth and development, including the development of the swim bladder. During early development, zebrafish undergo an embryonic-to-larval transition phase marking an important switch from yolk sac- to exogenous feeding larvae around 120 hpf. This transition includes swim bladder inflation, structural and functional maturation of the mouth and gastrointestinal tract, and resorption of the yolk sac [50]. Dong et al. [17] and Thisse et al. [31] showed localized expression of D1 and D2 in the swim bladder tissue of 96 and 120 hpf zebrafish larvae. Liu and Chan [50] showed that TH plays a major role in the transition phase and reported that inhibition of the TH-TR (thyroid hormone receptor) axis retarded swim bladder development. Furthermore, the swim bladder in fish is postulated as a homolog of the tetrapod lung [61]. Holt et al. [62] showed that hypothyroidism in neonatal rats also retarded the growth of the lung. The observed reduced growth and swim bladder inflation (Fig 6B and 6C) in D1D2MO zebrafish larvae was thus expected based on the previous evidence of hypothyroidism in D1D2MO since it could be rescued by T_3_ supplementation [14].

Our observation of similar (and even more prominent) effects on growth, development and swim bladder inflation in D3MO seems counterintuitive because these animals are expected to suffer from hyperthyroidism—confirmed by the previous finding that T_3_ supplementation partially mimicked the effects observed in D3MO [10]. Contradicting findings have been reported on the effects of T_3_ supplementation. While T_3_ supplementation was shown to improve swim bladder inflation, promote growth, differentiation and survival in some studies [63–65], it was shown in some other studies to impair swim bladder morphogenesis [10], delay hatching and inhibit growth and development [10,66] similar to what we observed. It can even cause swim bladder deflation possibly by suppressing surfactant production [50]. The reason for these contradicting results may be that the final effect on growth and development depends on the dose and the thyroid status of the organism, with a beneficial effect of a small increase in T_3_ levels but an adverse effect of T_3_ levels above physiological boundaries.

### 4. Summary

D3MO transiently increased the heart rate and probably resulted in an increased metabolic rate and a less efficient use of energy, contributing to reduced growth. A stimulation of gluconeogenesis, partly by using amino acids as a substrate could have led to the increased carbohydrate content and decreased protein content, also contributing to the reduced growth. Furthermore, transcripts related to muscle development were differentially expressed and motility was reduced. This was possibly also related to impaired swim bladder inflation and delayed hatching. The pronounced upregulation of a large number of transcripts involved in carbohydrate metabolism in D1D2MO could reflect a compensatory response to a decreased metabolic rate due to low T_3_ levels. Here, we also observed decreased motility, reduced protein content and differential expression of transcripts involved in muscle development. The transcriptional profile in the head was highly similar in both knockdowns and levels of transcripts involved in purine metabolism, phototransduction and eye development were decreased. These findings suggest that a fine-tuned TH balance is essential to coordinate vertebrate eye development and function and that D1D2 and especially D3 deficiency may impair eye development and function.

In conclusion, we have shown that the phenotype following knockdown of the TH activating or inactivating pathway shows several similarities, but the transcriptional patterns combined with the current knowledge of the role of thyroid hormones on vertebrate development indicate that they were caused through distinct molecular mechanisms. Zebrafish express different isoforms of TRs both for TRα (where there are even two genes) and for TRβ. Isoform-specific differences in ligand dependency of TR transactivation activity have been described and combined with tissue-dependent deiodinase expression this may contribute to the large difference in transcriptional expression pattern observed between the two knockdown conditions. Possibly the observed early phenotypic effects are common symptoms, but in a long-term scenario the phenotypes would further diverge due to these underlying differences.

## Supporting Information

S1 Table Summary of GO and KEGG analysis. The first worksheet shows all GO classes that reached the cutoff value, and shows how they were grouped into biological categories. The pie charts in Fig 1 are based on the scores of the biological categories (numbers in bold).
(XLSX)

S2 Table Differentially expressed transcripts. The first worksheet is a list of all differentially expressed transcripts with logFC and multiple testing corrected p values, and sheets 2–5 specifically show those transcripts shown in Figs 2–5. ‘#N/A’ indicates that a probe was not included in the analysis because it did not meet the spot filtering criteria. A logFC of ‘0’ indicates that the FDR was > 0.05 and therefore the transcript is not considered differentially expressed.
(XLSX)
